# Scabies-induced lichen simplex chronicus misdiagnosed as psychogenic pruritus: a case report

**DOI:** 10.1186/s13256-020-02628-x

**Published:** 2021-02-17

**Authors:** Ozzy M. Emmanuel, Ana V. Karovska, Okey Ikedilo

**Affiliations:** Maducare.inc, Chidicon Medical Center, Premier Medical Associates, The Villages, FL USA

**Keywords:** Lichen simplex, Psychogenic pruritus, Itch-scratch cycle, Burrows, Scabies, Sarcoptes

## Abstract

**Introduction:**

Lichen simplex chronicus has been defined as a localized skin condition characterized by thickening, hyperpigmentation and accentuated skin markings from chronic itching and from repeated scratching. The affected skin area is usually described as demarcated, and often circumscribed. It has even been defined as a “psychogenic pruritic disorder”. The idea of a neurological component has also been suggested, hence the term '*neurodermatitis circumscripta'.* However, the pathophysiology of this condition remains unclear. Several associations and etiologies have been reported in literature, including strong links with mental disorders—anxiety and obsessive compulsive disorder to be specific. We report this case, most importantly, to highlight the value of an open-minded approach to patients and the 'old-fashioned' physician character of empathy, the skill of detailed history taking and physical examination, and lastly to suggest that lichen simplex chronicus may not always present as a localized, '*circumscripta'* or demarcated area of skin.

**Case description:**

When a sixty-five year-old Caucasian female presented to our clinic agitated, intensely scratching her entire body and complaining of severe pruritus, an open-minded detailed approach during history taking and physical examination led to the working diagnosis of diffuse lichenification from chronic scratching secondary to a “possible” cutaneous disorder. Her medical history was unremarkable, but her psychiatric history was significant for Anxiety disorder. She remained on her anxiolytic medication. Her presenting symptom was reported to have persisted for more than 9 months. Review of previous unremarkable lab results and a remarkable findings on detailed skin inspection led to an empiric, trial regimen consisting of three topical preparations: an anti-pruritic—to break the itch-scratch cycle, anti-inflammatory—to curb any inflammatory/immune response and a 'last-ditch' scabicidal application. Follow-up was scheduled, but the patient called the office requesting an earlier follow up appointment. The lesions had significantly improved and the hyper-pigmented, indurated and escoriated skin appearance had resolved; and most importantly, the pruritus.

**Conclusion:**

Thus we conclude that lichen simplex chronicus may not always present as circumscribed or localized area of skin as currently noted in literature. Also, in patients with psychiatric conditions including anxiety and obsessive-compulsive disorder in particular, effort should be made to avoid stereotyping their presentation as part of their mental disorder spectrum. The value of detailed history and physical examination, mixed with empathy is highlighted. We make our recommendation considering the profound turnaround in the patient's condition and quality of life after several months of emotional and psychological suffering.

## Background

The presence of a pre-existing psychiatric diagnosis could pose a major barrier to proper evaluation, diagnosis and clinical care of patients presenting with somatic symptoms mixed with behavioral anomalies, even when the underlying etiology of their presentation may not be psychiatric at all. To make it even more challenging, already accepted definitions of disease entities could influence the provider's thought-process when interacting with patients, preventing them from exploring other possible differential diagnoses. In this report, we strongly believe that our patient with chronic skin lesions characterized by recalcitrant, non-remitting pruritus very likely suffered for so long because her lesions deviated from the widely accepted pattern of presentation for the disease condition. Hence our goal in this report is to highlight the importance of an open-minded approach to patient encounters. Lichen simplex chronicus, as defined in current literature, presents as a well-circumscribed, localized dermatological condition. There is also an accepted neurological association hence the term “neurodermatitis circumscripta” has also been used to describe the condition [1]. Also expressed and accepted in current literature is the fact the psychological factors could be the underlying cause for the condition, as noted in this statement: “Emotional tensions, such as in patients with anxiety, depression or obsessive compulsive disorder, may play a key role in inducing a pruritic sensation, leading to scratching that can become self-perpetuating” [2]. The condition has also boldly been described as a “psychogenic pruritic disorder” [3]. Thus, a patient presenting with severe generalized pruritus, accompanied with repeated scratching, agitation and other somatic symptoms, is very likely to be improperly evaluated and incorrectly diagnosed, particularly if the patient carries a preexisting diagnosis of psychiatric mood, personality or psychotic disorder.

The patient represented in this report had experienced significant physical and psychological trauma for several months due to chronic, pruritic skin lesions. However, with an open-minded, unbiased approach during the encounter and detailed physical examination, our team was able to arrive at the correct diagnosis and thus bring relief to the patient. Therefore, with this report, we intend to remind our audience once again that diseases may not always present as generally accepted in clinical literature, and the underlying etiology for a patient's symptoms may not always be psychogenic; hence the need for careful, detailed physical examination and empathy when interacting with patients.

## Case presentation

A 65 year-old Caucasian female presented to our outpatient clinic with repeated scratching and generalized pruritus, with visible areas of hyperpigmentation, hyperkeratosis, induration and extensive excoriations on her skin. This was reported to have lasted for about 9–10 months. She denied any definitive precipitating factors. She also denied any previous skin lesions or conditions prior to the current lesions. She did not recall exposure to any toxic substances or chemicals. Patient also denied use of any new cosmetic products. There was no zoonotic contacts reported or any exposures to chemicals or new cosmetic products prior to the appearance of the lesions. She denied any significant change in her health status, except an uneventful elective laparoscopic cholecystectomy few months earlier, with good recovery. Her medical history was otherwise unremarkable and her surgical history was as mentioned above. Of note, her psychiatric history was significant for a diagnosis of anxiety disorder, for which she had been managed by several Psychiatrists and remained on her anxiolytic medication, Effexor. Her social history was negative for tobacco, alcohol or recreational drug use and her family history was unremarkable and non-contributory. She disclosed cohabiting with her spouse with whom she maintained a monogamous relationship in a close-knit community for the elderly. On presentation, the patient was in obvious state of anxiety and agitation but neither combative nor violent.

## Previous management

During the interview, the patient disclosed previous consultation and management by two different dermatologists. Skin biopsy by one of the dermatologists came back as “non-definitive” and subsequent genetic testing was reported as 'inconclusive”. A copy of this report was reviewed during the visit to our clinic. Per patient, she had also undergone previous treatment with a scabicidal topical preparation—Permethrin (*x*2) but her symptoms remained refractory. Her psychiatrist then focused on managing her anxiety and, after several visits, discharged with the suggestion that her symptoms were very likely “all in her head”.

## Physical examination

**Fig. 1:**
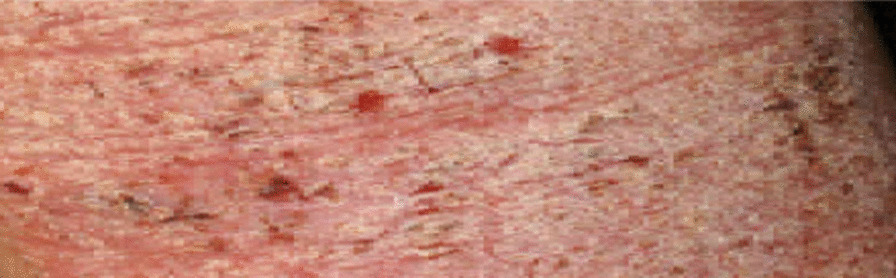
Image representation of patient's skin lesions prior to trial treatment regimen

**Fig. 2:**
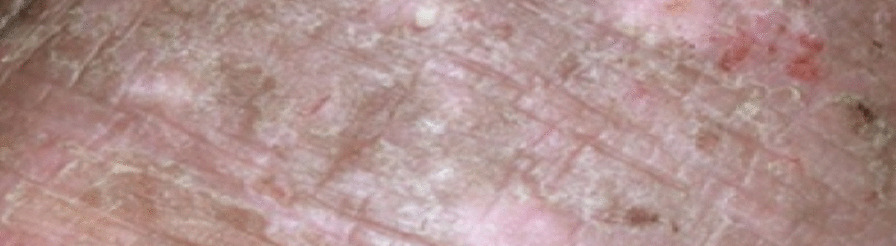
Image representation of patient's skin lesions prior to trial treatment regimen

On general examination, the patient was well developed, well nourished female in good physical state compared to her stated age. She appeared well kept with her body weight, BMI and vital signs obtained during the visit were within normal limits for her age. Cardiac, pulmonary and musculoskeletal examination also did not reveal any remarkable abnormalities. Neuropsychiatric evaluation was negative for any focal neurological deficits but remarkable for severe anxiety, panic and marked restlessness as she profusely and repeatedly scratched her entire body. She was alert and oriented x4. She denied homicidal and suicidal ideations at the time but disclosed past episodes of thoughts of self-harm self-harm due to exacerbation of her symptoms. Integumentary examination revealed extensive lichenification, hyperpigmentation, thickening and skin induration with several excoriation marks. The areas of lichenification and hyperpigmentation were not clearly circumscribed. Instead, there was diffuse skin involvements including both upper and lower limbs, neck and upper back. *(representation of the lesions in* Figs. 1 and 2). Facial and anterior abdominal regions were spared from lichenification but there were visible “scratch marks” on her abdomen. Remarkably, her spouse had not demonstrated any such lesions over the past 9–10 months, but patient recalled that he (her husband) had just recently developed few 'itchy', tiny blister-like lesions in the web of his fingers, and those blisters itched more at night. That piece of information was considered vital; and we decided to re-examine the patient's skin more closely driven mostly by an unbiased thought process and empathy. This closer, more detailed inspection eventually revealed minimally visible tiny “tracks” or “burrows” which we suspected must be tracks created by sarcoptes scabiei mites. With that, we effectively and most importantly ruled out the idea of a psychogenic etiology and temporarily, other possible etiologies including eczema.

## Empiric treatment

Patient was treated empirically with a trial regimen consisting of three different topical applications: an anti-pruritic preparation 'extra strength' Benadryl—to break to itch-rub-scratch cycle, a mid-high potency topical anti-inflammatory ointment, triamcinolone acetonide—to inhibit the inflammatory cascade and lastly, topical Lindane application—with precautionary measures taken (monitor for hepatotoxity). Previously used medications were avoided considering the possibility of developing tolerance and the absence of any remarkable improvement reported with the previous medication. Specific instruction was also given to the patient considering the medication's known potential adverse effects, as well as the mites' mode of spread which could lead to re-infestation. Follow-up was scheduled in 2 weeks to re-evaluate and re-assess the patient's condition and response, if any, to the trial therapy.

## Result

Patient called to schedule her follow-up visit much earlier than the scheduled follow up date. Her skin lesions, induration, hyperpigmentation, thickening and excoriations had resolved. Her mood and affect was remarkably different from her initial visit. Patient demonstrated no signs of anxiety or agitation. The pruritus and repeated scratching had resolved. The outcome was so remarkable she permitted everyone to see the improvement in her skin lesions. For our team, we anticipated some improvement but the result was so impressive we thought it would be valuable to report this case and share what we learned with others in the clinical community. Consent was given by the patient to report this case after detailed discussion regarding the educative value of reporting this case.

## Discussion

The value of detailed, skilled and empathetic approach to patient encounters and physical examination remains undisputed. However, several factors can pose a challenge to a proper open-minded approach when dealing with patients. Some of these include current definitions and descriptions of a disease condition, as well as pre-existing diagnoses carried by the patients. The patient in this case presented with diffuse hyperpigmented, hyperkeratotic skin with induration and significant thickening which on visual inspection and manual examination was determined to be chronic lichenification, most likely due to the repeated intense scratching. In patients with pre-existing psychiatric diagnoses, such somatic symptoms could impede proper, detailed physical examination and subsequent accurate diagnosis and management.

Having been finally informed by her Psychiatrist that her symptom was “all in her head”, we reasoned differently considering visible abnormalities on her skin. Our most important goal became to uncover any possible underlying dermatological etiology for such recalcitrant pruritus and scratching. Again, since we did not wish to assume the patient's Psychiatric care, we deferred any extensive psychiatric assessments, focusing instead on extensive physical examination. Previous lab results were reviewed including CBC, CMP, inflammatory and immunoserolgic markers, as well as a copy of the genetic testing report which the patient brought to the clinic - all of which were unremarkable. Our approach was to remain very open-minded, thorough and above all, empathetic. Thus we were able to obtain the single-most valuable information during the encounter which was the pruritic, tiny, blister-like lesions in the web of her spouse's fingers. According to the patient, this was new-onset, as her spouse had remained asymptomatic for the previous eight months. Again of note: the pruritus was exacerbated nocturnally.

With sarcoptes scabiei infestation high on our differential diagnoses list, detailed closer skin inspection confirmed the diagnosis of scabies—typically a clinical diagnosis. The clinical outcome was remarkably impressive as already mentioned. Plan was also put in place to initiate oral therapy with Ivermectin should the topical trial regimen fail. Eventually that was not necessary. With regards to possible source of exposure and infestation, patient could not clearly recall any specific sources, although we believe there could have been some level of immuno-suppression following her elective surgery which could have played a role in the infestation. In this report therefore, we propose that the diagnosis of scabies-induced lichen simplex chronicus was missed due to the patient's pre-existing psychiatric diagnosis of anxiety disorder. We also believe the definition of lichen simplex chronicus in current literature as “circumscribed, well demarcated” skin condition impeded proper evaluation and diagnosis of the underlying cause of her chronic pruritus and scratching.

We decided to report this case to highlight and emphasize the value of in-depth and detailed physical examination especially in patients with known mental disorders, to also suggest that lichen simplex chronicus may not always present as well-demarcated, circumscribed lesions, in contrast to current literature, and above all to emphasize the importance of detailed, unbiased approach to patient encounters and physical examination.

## Conclusion

In conclusion, we propose that although it is generally accepted that 'psychological factors *may* contribute to both the development and persistence of Lichen Simplex Chronicus [4], we still encourage an unbiased and detailed approach to history taking and physical examination. We make this recommendation since we believe the patient's pre-existing diagnosis of anxiety disorder negatively influenced the thought process of her previous health care providers, until an open-minded approach and detailed physical examination by the attending Physician helped uncover the cause of the patient's suffering.

Secondly, we suggest that lichen simplex chronicus may not always present as '*circumscripta'* or localized, demarcated skin lesion as currently presented in literature till date. Thus we recommend a modification of the current definition of Lichen Simplex Chronicus to include the possibility of diffuse dermatological involvement. We also wish to remind our readers of the priceless value of the 'tried and trusted' physician character of empathy which will ensure that genuine effort is made to get to the root of a patient's presenting symptoms.

Finally and most importantly, in patients with mental disorders, effort should be made to avoid stereotyping their presentation as a “regular” component of their disease spectrum. Detailed, extensive, empathetic and an open-minded approach to patient interviews and physical examination should be implemented regardless of a patient's pre-existing psychiatric diagnoses. Otherwise, we may find ourselves generalizing and waiving off these (sometimes) helpless but precious patients who come to us needing care and solutions to their health problems.

## Data Availability

Data sharing is not applicable to this article as no data-sets were generated or analyzed in this report.

## Confidentiality

Appropriate measures have been taken to ensure confidentiality and to protect patient's identification.
